# A low component count high step up DC–DC converter using a coupled inductor

**DOI:** 10.1038/s41598-026-55302-5

**Published:** 2026-05-28

**Authors:** Ali Paşaoğlu, Ali Nadermohammadi, Hamed Shams, Sara Pournaji İran

**Affiliations:** 1 Computer Engineering Department, Engineering and Natural Science Faculty, İstanbul Rumeli University, İstanbul, Turkey; 2 Faculty of Electrical and Computer Engineering, University of Tabriz, Tabriz, 51666-16471 Iran; 3 Management Information Systems Department, Faculty of Economics, Administrative and Social Sciences, İstanbul Rumeli University, İstanbul, Turkey

**Keywords:** DC–DC converter, High step-up, Coupled inductor, Renewable energy, Low input-current ripple, Energy science and technology, Engineering

## Abstract

This paper introduces a non-isolated DC–DC boost converter specifically designed for renewable-energy applications, emphasizing low input-current ripple and a reduced number of components. The topology incorporates a coupled inductor with two windings, allowing the converter to achieve very high voltage step-up ratios at relatively modest duty cycles. Operating at lower duty ratios helps decrease conduction losses in the main switch and contributes to improved overall efficiency. The voltage conversion gain of the proposed high-step-up converter can be readily adjusted through two independent design parameters: the duty cycle of the primary switching device and the turns ratio of the coupled-inductor windings. This dual degree of freedom provides considerable design flexibility for meeting diverse application requirements. The proposed converter offers several notable benefits, including extremely high voltage amplification, reduced voltage stress on power semiconductor devices, a continuous input current profile, and a shared ground reference between the input source and the load. Furthermore, the use of a minimal number of components particularly semiconductor devices enhances efficiency and simplifies implementation. The topology also employs synchronized switching, which streamlines the control strategy and further improves performance. A detailed analysis of the converter’s operating principle is presented, covering its various switching modes. To demonstrate its advantages, the proposed design is systematically compared with conventional high-gain converter topologies. Experimental verification is carried out using a 400 W laboratory prototype operating at a switching frequency of 50 kHz, which successfully steps up a 29 V input to a regulated 400 V output, thereby validating both the analytical results and the practical feasibility of the converter.

## Introduction

Renewable energy sources (RES), such as photovoltaic modules and fuel cells, inherently deliver electrical power at relatively low DC voltage levels, typically below 50 V. To make this power usable in practical systems, high step-up (high-gain) DC–DC converters are required to elevate the voltage to appropriate levels. In addition to renewable energy interfaces, step-up converters are extensively used in a wide range of applications, including medical devices, energy harvesting systems, DC microgrids, lighting installations, and portable electronics^1–4^. As a result, switched-mode DC–DC converter technologies have become a major focus of ongoing research efforts. For RES-based systems in particular, maintaining a continuous and non-pulsating input current is crucial for maximizing energy extraction, making current-fed converter configurations especially attractive^5–8^.

To address the need for high voltage amplification, numerous modified DC–DC converter topologies have been proposed to boost low-voltage inputs to high DC output levels^9,10^. These designs employ a variety of voltage-enhancement techniques, such as switched-inductor and switched-capacitor structures, cascaded stages, interleaving, magnetic coupling, voltage multiplier (VM) cells, and hybrid combinations of these methods^11–22^. Although effective in increasing voltage gain, many of these approaches suffer from practical drawbacks in high-gain applications, primarily due to their large component count and reliance on hard-switching operation. These factors increase circuit complexity, elevate power losses, and ultimately degrade efficiency.

Magnetic-based solutions, including coupled inductors (CIs) and transformers often combined with VM networks offer another effective means of extending voltage gain. In such configurations, both the duty cycle and the turns ratio provide additional degrees of freedom, enabling a wider range of voltage conversion^23^. However, CI-based converters are inherently affected by leakage inductance, which can produce high-voltage spikes across the power switches. To suppress these transients, additional active or passive clamping circuits are generally required, increasing circuit complexity and cost^24^.

An alternative strategy for achieving large voltage gains involves the use of multi-stage or multi-level converter structures, which are commonly classified into cascaded, interleaved, and multilevel topologies^25^. While these architectures are effective in boosting voltage, they typically demand complex control schemes, involve a high number of components, and result in increased system cost. Interleaved converters, in particular, have been widely explored for photovoltaic applications due to their ability to reduce input current ripple^26^. Despite this advantage, their practical implementation is often limited by sophisticated control requirements and higher hardware complexity.

Among the many available DC–DC converter configurations, quadratic converters have attracted considerable attention because they can achieve high voltage gain with relatively few components^27,28^. In these topologies, the voltage conversion ratio increases nonlinearly with the duty cycle, enabling large step-up ratios. In recent years, several non-isolated quadratic converters capable of high voltage gain have been reported^29,30^. For example, a transformerless single-switch quadratic boost converter was introduced in^31^ for low-voltage applications. Although capable of achieving substantial voltage amplification, such designs often rely on a large number of components, which limits their practicality. Moreover, they typically exhibit significant input current ripple, making them less suitable for RES applications.

To mitigate this issue, single-switch quadratic DC–DC converters based on coupled inductors with reduced input current ripple were proposed in^32,33^. However, these designs suffer from considerable power losses in their input diodes. Similarly, alternative quadratic step-up converters with reduced switch voltage stress were presented in^34,35^. While these topologies can achieve high voltage gain, the direct series connection between the CI primary winding and the input source leads to increased input current ripple. More recent approaches have employed three-winding coupled inductors (TWCIs) combined with VM cells to provide additional degrees of freedom in controlling the voltage gain^36^. Despite their flexibility, these designs still face major challenges, including high voltage stress on the power switch and elevated current stress in the input diodes. Additionally, several quadratic CI-based converters proposed in^37,38^ are limited by their relatively modest achievable voltage gain.

Motivated by the strengths of existing high step-up converters while addressing their shortcomings, this paper introduces a new quadratic high step-up DC–DC converter topology. The proposed design offers several notable advantages. First, it provides two independent control parameters the duty cycle of a single power switch and the turns ratio of the coupled inductor allowing flexible and precise regulation of the voltage gain. Unlike traditional Z-source (ZS) and quasi-Z-source (QZS) converters, which restrict the duty cycle to the range of 0–0.5, the proposed topology permits duty-cycle variation from 0 to 1, enabling higher voltage gains without limitation. Second, the inclusion of soft-switching operation significantly reduces switching losses, thereby enhancing efficiency. Third, the voltage stress across the switch and diodes is substantially reduced, allowing the use of low-voltage-rated devices with smaller on-state resistance, which further lowers conduction losses. Finally, the converter is capable of delivering high output voltage even at low duty cycles, minimizing conduction losses and resulting in improved overall system efficiency.

## Proposed topology and operation modes

**Fig. 1:**
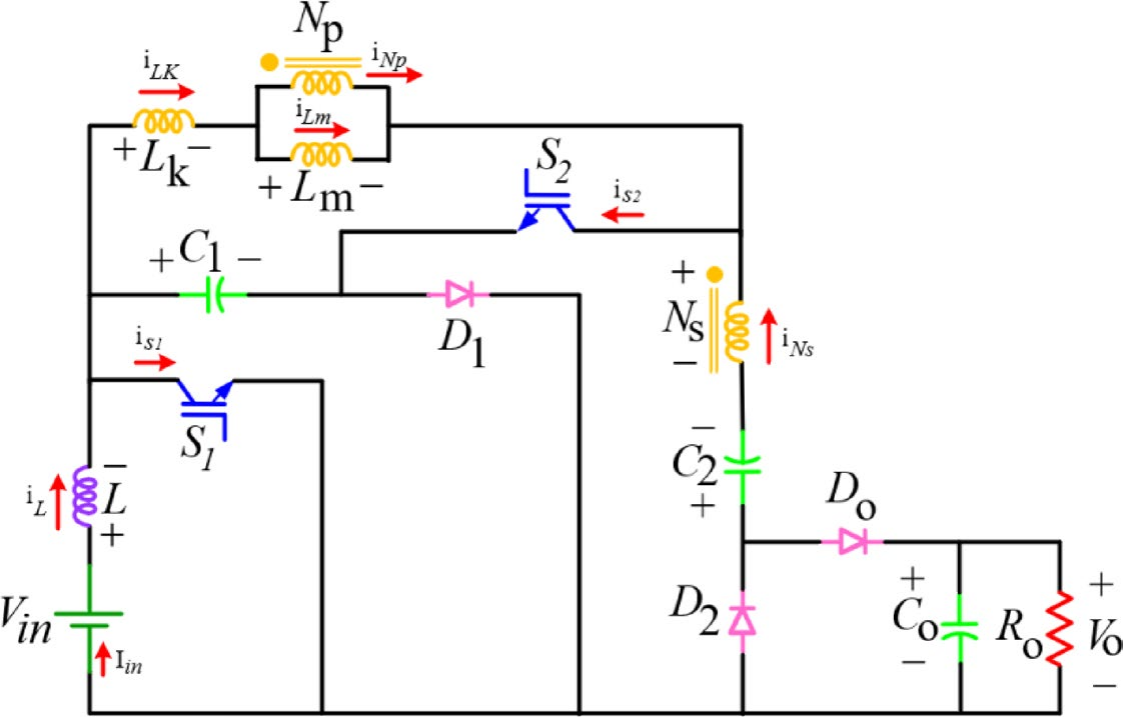
Power circuit of the suggested configuration.

**Fig. 2:**
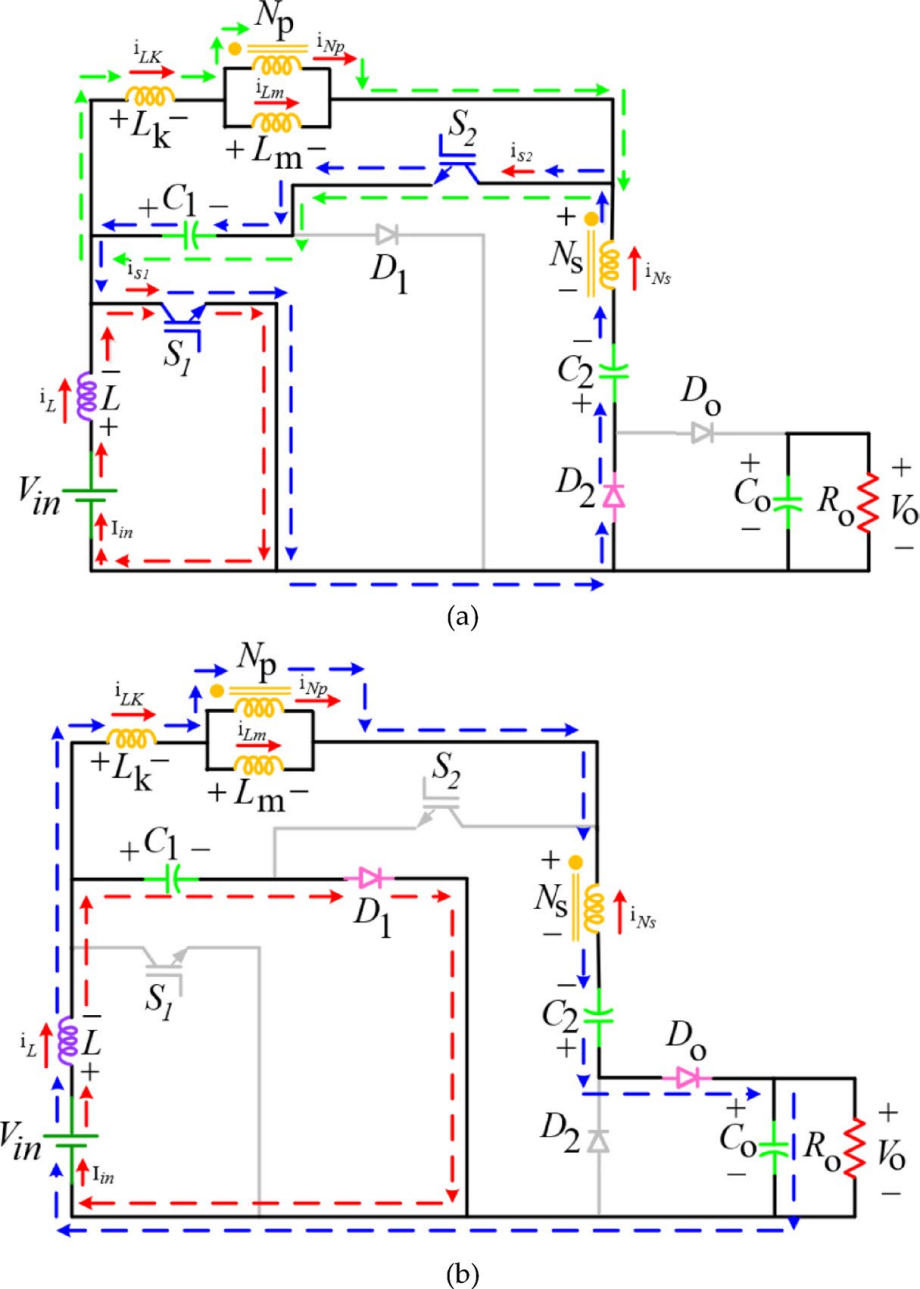
Equivalent representations of the suggested converter during boost-mode operation: (**a**) initial switching interval, and (**b**) subsequent switching interval.

**Fig. 3:**
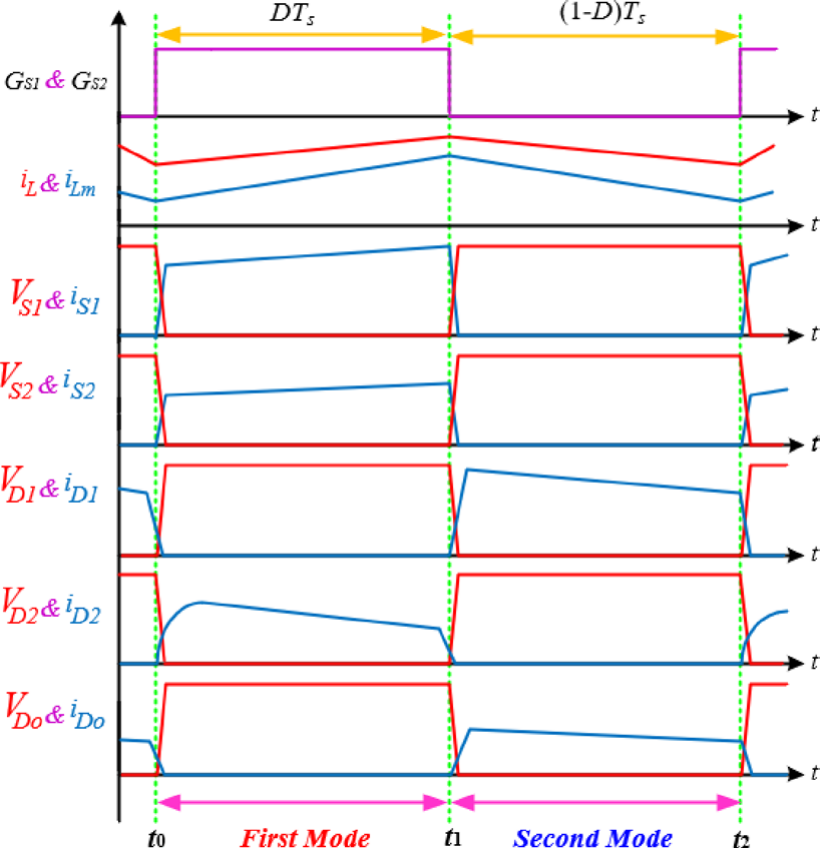
Main waveforms of the proffered structure.

Figure 1 depicts the overall configuration of the proposed converter topology. The circuit is composed of two active switching devices (S₁ and S₂), three capacitors (C₁, C₂, and C₀), three diodes (D₁, D₂, and D₀), a single input inductor (L), and a coupled inductor formed by two magnetically linked windings. These windings include a primary coil (Nₚ) and a secondary coil (Nₛ), whose relationship is characterized by the turns ratio n = N_s_/N_p_. The degree of magnetic coupling between the two windings is expressed by the coupling coefficient k = L_m_/(L_m_+L_k_). When the proposed high voltage gain converter operates in continuous conduction mode (CCM), one complete switching period can be segmented into three distinct operating states, as illustrated in Fig. 2. The associated voltage and current waveforms of the key circuit elements for these operating intervals are shown in Fig. 3.

### First mode

During this interval of operation, both power switches are in the ON state, which forward-biases diode D₂ while the remaining diodes are reverse-biased. Under these conditions, the magnetizing inductance L_m_ is energized through the conduction path formed by C₁–L_m_–S₂. As a result, the magnetizing current (i_Lm_) increases linearly, accompanied by the discharge of capacitor C₁ along this loop. In parallel, the input inductor L is charged through the V_in_–L–S₁ path, causing the input current (i_L_) to rise linearly. At the same time, capacitor C₂ accumulates energy through the conduction loop consisting of D₂, C₂, N_S_, S₂, C₁, and S₁. Based on these operating conditions, the governing equations for this interval can be established as follows.

1 $${V_{Ns}}=n{V_{Lm}}$$

2 $${V_1}={V_{Lm}}+{V_{Lk}}=\frac{{{V_{Lm}}}}{k}$$

3 $${V_{in}}={V_L}$$

4 $${V_{Ns}}={V_{C2}} - {V_{C1}}$$

5 $${V_1}={V_{C1}}$$

6 $${I_{in}}={I_L}$$

7 $${I_{S1}}={I_L} - {I_{C1}} - {I_{Lm}} - {I_{Np}}$$

8 $${I_{S2}}= - {I_{C1}}$$

9 $${I_{Lm}}= - {I_{Np}} - {I_{Ns}}+{I_{S2}}$$

10 $${I_{C2}}={I_{Ns}}$$

11 $${I_{C2}}={I_{D2}}$$

12 $${I_{Co}}= - {I_O}$$

13 $${I_{Co}}= - {I_O}+{I_{D2}} - {I_{S1}}+{I_{in}}$$

### Second mode

In this stage of operation, the power switch S is switched off, causing diodes D_2_, D_3_, D_4_, and D_5_ to become forward-biased, while the other diodes remain non-conducting. During this interval, the currents flowing through both the input inductor and the magnetizing inductor decrease in a nearly linear manner. At the same time, capacitors C_1_–C_4_ are charged, whereas the output capacitor C_O_ releases energy to the load. The governing voltage and current relationships for this mode of operation can be expressed.

14 $${V_{in}} - {V_L}={V_{C1}}$$

15 $$- {V_{C1}}+{V_1}+{V_{Ns}}+{V_O}={V_{C2}}$$

16 $${I_{D1}}={I_{C1}}$$

17 $${I_{Ns}}= - {I_{Lm}} - {I_{Np}}$$

18 $${I_{C1}}={I_L} - {I_{Np}} - {I_{Lm}}$$

19 $${I_{C2}}={I_{Ns}}$$

20 $${I_{C2}}= - {I_{Do}}.$$

21 $${I_{Co}}={I_{Do}} - {I_O}$$

22 $${I_{Co}}= - {I_O} - {I_{D1}}+{I_{in}}.$$

### Voltage gain calculation

The steady-state characteristics of the proposed converter operating in continuous conduction mode are investigated by evaluating the circuit behavior during each switching interval and examining the associated equivalent circuits, as shown in Fig. 2. By enforcing the volt–second balance condition on both the input inductor and the magnetizing inductance, the voltages across the capacitors can be derived, along with the mathematical expression that relates the input voltage to the output voltage. Considering the individual operating states, the following analytical relations are obtained.

23 $${V_{C1}}=\frac{{{V_{in}}}}{{1 - D}}=58({\mathrm{V}})$$

24 $${V_{C2}}=\frac{{{V_{in}}(kn+1)}}{{1 - D}}=174({\mathrm{V}})$$

The analytical formulation describing the output voltage of the proposed converter is expressed as follows:

25 $${V_O}=\frac{{{V_{in}}(kn - D+2)}}{{{{(1 - D)}^2}}}=406({\mathrm{V}})$$

Assuming ideal magnetic coupling in the coupled inductor (k = 1), the expression for the output voltage gain can be written as:

26 $$G=\frac{{{V_O}}}{{{V_{in}}}}=\frac{{n - D+2}}{{{{(1 - D)}^2}}}=14$$

### Voltage stresses of semiconductors

The voltage allocation across the power switches and diode devices can be expressed as follows:

27 $${V_{S1}}=\frac{{{V_{in}}}}{{1 - D}}=58({\mathrm{V}})$$

28 $${V_{S2}}=\frac{{{V_{in}}}}{{{{(1 - D)}^2}}}=116({\mathrm{V}})$$

29 $${V_{D1}}=\frac{{{V_{in}}}}{{1 - D}}=58({\mathrm{V}})$$

30 $${V_{D2}}={V_{Do}}=\frac{{{V_{in}}(kn - D+2)}}{{{{(1 - D)}^2}}}=406({\mathrm{V}})$$

### Current analysis of the components

Based on the operating waveforms shown in Fig. 2 and the corresponding analytical expressions, the current characteristics of the switches and diodes in each operating mode can be described as follows:

31 $${I_{S1 - Mode1}}=\frac{{{I_O}(kn - n+Dn+1)}}{{D{{(1 - D)}^2}}}=16({\mathrm{A}})$$

32 $${I_{S2 - Mode1}}=\frac{{{I_O}(kn+1)}}{{D(1 - D)}}=12({\mathrm{A}})$$

33 $${I_{D1 - Mode2}}=\frac{{{I_O}(kn+1)}}{{{{(1 - D)}^2}}}=12({\mathrm{A}})$$

34 $${I_{D2 - Mode1}}=\frac{{{I_O}}}{D}=2({\mathrm{A}})$$

35 $${I_{Do - Mode2}}=\frac{{{I_O}}}{{1 - D}}=2({\mathrm{A}})$$

The analytical expressions used to determine the currents flowing through inductors L and L_m_ are given below:

36 $${I_{in}}={I_L}=\frac{{{I_O}(kn - D+2)}}{{{{(1 - D)}^2}}}=14({\mathrm{A}})$$

37 $${I_{Lm}}=\frac{{{I_O}(1+n)}}{{1 - D}}=6({\mathrm{A}})$$

By examining the equivalent circuits corresponding to each operating interval and applying the ampere–second balance condition, the following expressions for the capacitor currents are derived:

38 $${I_{C1 - Mode1}}= - \frac{{{I_O}(kn+1)}}{{D(1 - D)}}= - 12({\mathrm{A}})$$

39 $${I_{C1 - Mode2}}=\frac{{{I_O}(kn+1)}}{{{{(1 - D)}^2}}}=12({\mathrm{A}})$$

40 $${I_{C2 - Mode1}}=\frac{{{I_O}}}{D}=2({\mathrm{A}})$$

41 $${I_{C2 - Mode2}}= - \frac{{{I_O}}}{{1 - D}}= - 2({\mathrm{A}})$$

42 $${I_{Co - Mode1}}= - {I_O}= - 1({\mathrm{A}})$$

43 $${I_{Co - Mode2}}=\frac{{D{I_O}}}{{1 - D}}=1({\mathrm{A}})$$

### Average currents of semiconductors

The average current stresses experienced by the power switch and diode components can be expressed as follows:

44 $${I_{S1,avg}}=\frac{{{I_O}(kn - n+Dn+1)}}{{{{(1 - D)}^2}}}=8({\mathrm{A}})$$

45 $${I_{S2,avg}}=\frac{{{I_O}(kn+1)}}{{(1 - D)}}=6({\mathrm{A}})$$

46 $${I_{D1,avg}}=\frac{{{I_O}(kn+1)}}{{1 - D}}=6({\mathrm{A}})$$

47 $${I_{D2,avg}}={I_{Do,avg}}={I_O}=1({\mathrm{A}})$$

An accurate assessment of power losses in power converters requires the calculation of the root-mean-square (RMS) currents for all circuit components. The methods used to determine the RMS currents of each element are presented in Eqs. (48)–(55), as outlined below:

48 $${I_{S1,RMS}}=\frac{{{I_O}(Dn - n+kn+1)}}{{\sqrt D {{(1 - D)}^2}}}=11.31({\mathrm{A}})$$

49 $${I_{S2,RMS}}=\frac{{{I_O}(kn+1)}}{{\sqrt D (1 - D)}}=8.48({\mathrm{A}})$$

50 $${I_{D1,RMS}}=\frac{{{I_O}(1+kn)}}{{\sqrt {{{(1 - D)}^3}} }}=8.48({\mathrm{A}})$$

51 $${I_{D2,RMS}}=\frac{{{I_O}}}{{\sqrt D }}=1.41({\mathrm{A}})$$

52 $${I_{Do,RMS}}=\frac{{{I_O}}}{{\sqrt {1 - D} }}=1.41({\mathrm{A}})$$

53 $${I_{C1,RMS}}=\frac{{{I_O}(kn+1)}}{{\sqrt D (1 - D)}}+\frac{{{I_O}(kn+1)}}{{\sqrt {{{(1 - D)}^3}} }}=16.97({\mathrm{A}})$$

54 $${I_{C2,RMS}}==\frac{{{I_O}}}{{\sqrt D }}+\frac{{{I_O}}}{{\sqrt {(1 - D)} }}=2.82({\mathrm{A}})$$

55 $${I_{Co,RMS}}={I_o}\sqrt D +\frac{{{I_O}D}}{{\sqrt {1 - D} }}=1.4({\mathrm{A}})$$

### Efficiency calculation

This section focuses on evaluating both conduction and switching losses of the proposed converter in order to improve its overall efficiency. The analysis accounts for the influence of non-ideal characteristics, such as the intrinsic resistances of the diodes (r_D_), the power switch (r_S_), the input inductor (r_L_), the coupled inductor’s magnetizing branch (r_Lm_), and the capacitors (r_C_), as well as the forward voltage drops of the diodes (V_FD_) and the switch (V_FS_). Using these parameters, the expressions governing conduction and switching losses are derived as follows:

56 $$\left\{ \begin{gathered} {P_{Conduction,Switches}}=\sum\limits_{{n=1}}^{2} {{\mathrm{(}}{{\mathrm{V}}_{{\mathrm{FSn}}}}} \times {{\mathrm{I}}_{Sn - average}})+({r_{Sn}} \times {{\mathrm{I}}_{Sn - RMS}}^{2}) \hfill \\ {P_{Conduction,Switches}}={P_{Cond,{S_1}}}+{P_{Cond,{S_2}}}=3.55+3.06=6.61({\mathrm{W}}) \hfill \\ \end{gathered} \right.$$

57 $$\left\{ \begin{gathered} {P_{Conduction,{D_1}}}=({{\mathrm{V}}_{{\mathrm{F}}{{\mathrm{D}}_1}}} \times {{\mathrm{I}}_{{D_1} - average}})+({r_{{D_1}}} \times {{\mathrm{I}}_{{D_1} - RMS}}^{2})=3.65({\mathrm{W}}) \hfill \\ {P_{Conduction,{D_2}}}=({{\mathrm{V}}_{{\mathrm{F}}{{\mathrm{D}}_2}}} \times {{\mathrm{I}}_{{D_2} - average}})+({r_{{D_2}}} \times {{\mathrm{I}}_{{D_2} - RMS}}^{2})=0.51({\mathrm{W}}) \hfill \\ {P_{Conduction,{D_O}}}=({{\mathrm{V}}_{{\mathrm{F}}{{\mathrm{D}}_O}}} \times {{\mathrm{I}}_{{D_O} - average}})+({r_{{D_O}}} \times {{\mathrm{I}}_{{D_O} - RMS}}^{2})=1.2({\mathrm{W}}) \hfill \\ \end{gathered} \right.$$

58 $$\left\{ \begin{gathered} {P_{Conduction,L}}=({r_L} \times {{\mathrm{I}}_L}^{2})=1.14({\mathrm{W}}) \hfill \\ {P_{Conduction,Lm}}=({r_{Lm}} \times {{\mathrm{I}}_{Lm}}^{2})=0.2({\mathrm{W}}) \hfill \\ \end{gathered} \right.$$

59 $$\left\{ \begin{gathered} {P_{Conduction,{C_1}}}=({r_{{C_1}}} \times {{\mathrm{I}}_{{C_1} - RMS}}^{2})=2.79({\mathrm{W}}) \hfill \\ {P_{Conduction,{C_2}}}=({r_{{C_2}}} \times {{\mathrm{I}}_{{C_2} - RMS}}^{2})=0.07({\mathrm{W}}) \hfill \\ {P_{Conduction,{C_O}}}=({r_{{C_O}}} \times {{\mathrm{I}}_{{C_O} - RMS}}^{2})=0.019({\mathrm{W}}) \hfill \\ \end{gathered} \right.$$

60 $$\left\{ \begin{gathered} {P_{Switching,Switches}}=\sum\limits_{{n=1}}^{2} {{\mathrm{(}}\frac{1}{6} \times {f_{\mathrm{s}}} \times {{\mathrm{V}}_{{\mathrm{Sn}}}}} \times {{\mathrm{I}}_{Sn}} \times ({t_{ON}}+{t_{OFF}})) \hfill \\ {P_{Switching,Switches}}={P_{SW,{S_1}}}+{P_{SW,{S_2}}}=0.82+0.13=0.95({\mathrm{W}}) \hfill \\ \end{gathered} \right.$$

61 $$\left\{ \begin{gathered} {P_{Switching,{D_1}}}=\frac{1}{6} \times {f_{\mathrm{s}}} \times {{\mathrm{V}}_{{D_1}}} \times {I_{rr}} \times {t_b}=1.29 \times {10^{ - 5}}({\mathrm{W}}) \hfill \\ {P_{Switching,{D_2}}}=\frac{1}{6} \times {f_{\mathrm{s}}} \times {{\mathrm{V}}_{{D_2}}} \times {I_{rr}} \times {t_b}=9.06 \times {10^{ - 5}}({\mathrm{W}}) \hfill \\ {P_{Switching,{D_O}}}=\frac{1}{6} \times {f_{\mathrm{s}}} \times {{\mathrm{V}}_{{D_O}}} \times {I_{rr}} \times {t_b}=9.06 \times {10^{ - 5}}({\mathrm{W}}) \hfill \\ \end{gathered} \right.$$

The total power dissipation resulting from both conduction and switching losses in the switches and diodes is expressed as follows:

62 $${P_{S,Tot}}={P_{Cond,S1}}+{P_{SW,S1}}+{P_{Cond,S2}}+{P_{SW,S2}}=7.57({\mathrm{W}})$$

63 $${P_{D,Tot}}={P_{Cond,{D_{1,2,O}}}}+{P_{SW,{D_{1,2,O}}}}=5.36({\mathrm{W}})$$

The formula used to calculate the overall power losses in the capacitors is given as:

64 $${P_{C,Tot}}={P_{Cond,{C_1}}}+{P_{Cond,{C_2}}}+{P_{Cond,{C_O}}}=2.89({\mathrm{W}})$$

The power loss associated with the inductor core is determined using the following expression:

65 $${P_C}=kf_{s}^{\alpha }B_{m}^{\beta }$$

Inductor core losses (P_C_) are typically characterized in units of watts per kilogram (W/kg). These losses are commonly estimated using the Steinmetz formulation, which relies on three experimentally derived coefficients α, β, and k collectively known as the Steinmetz parameters. For a specific magnetic material, these parameters are usually supplied by the core manufacturer. In ferrite-based magnetic cores, the exponent α generally falls within the range of 1 to 2. By applying Faraday’s law, the resulting expression for estimating the magnetic core loss of the inductor can be written as follows:

66 $${V_L}=N\frac{{d\varphi (t)}}{{dt}}=N{A_c}\frac{{dB(t)}}{{dt}}$$

The parameter Ac represents the effective cross-sectional area of the magnetic core, a value typically provided by the core manufacturer. Based on this parameter, the maximum variation in magnetic flux density (ΔB) for the inductors can be defined as follows:

67 $$\Delta B=\frac{1}{{N{A_c}}}\int\limits_{0}^{{D{T_s}}} {{V_{in}}dt}$$

For inductive components, the overall core power loss is obtained by multiplying the specific core loss by the magnetic core mass, expressed as P_Core_=PC M, where M denotes the mass of the core material. Assuming that the maximum flux density is given by B_m_ = ΔB/2, the resulting formulation for estimating the core loss can be written as follows:

68 $${P_{Core}}=kf_{s}^{\alpha }{\left( {\frac{{{V_{in}}D}}{{2{N_P}{A_c}{f_s}}}} \right)^\beta }M=2 \times 0.718=1.43({\mathrm{W}})$$

An ETD 49/25/16 magnetic core is chosen for the inductors, with Steinmetz parameters specified as k = 4.124 × 10^− 5^, α = 1.72, and β = 2.76. Accordingly, the total power losses of the proposed high step-up converter can be expressed as follows:

69 $${P_{Loss}}={P_{S,Tot}}+{P_{D,Tot}}+{P_{Cond,Lm}}+{P_{Cond,L}}+{P_{Core}}+{P_{C,Tot}}=18.6297({\mathrm{W}})$$

Accordingly, the overall efficiency (η) of the proposed converter is determined as follows:

70 $$\eta =\frac{{{P_{Out}}}}{{{P_{Out}}+{P_{Loss}}}}=95.55\%$$

**Fig. 4:**
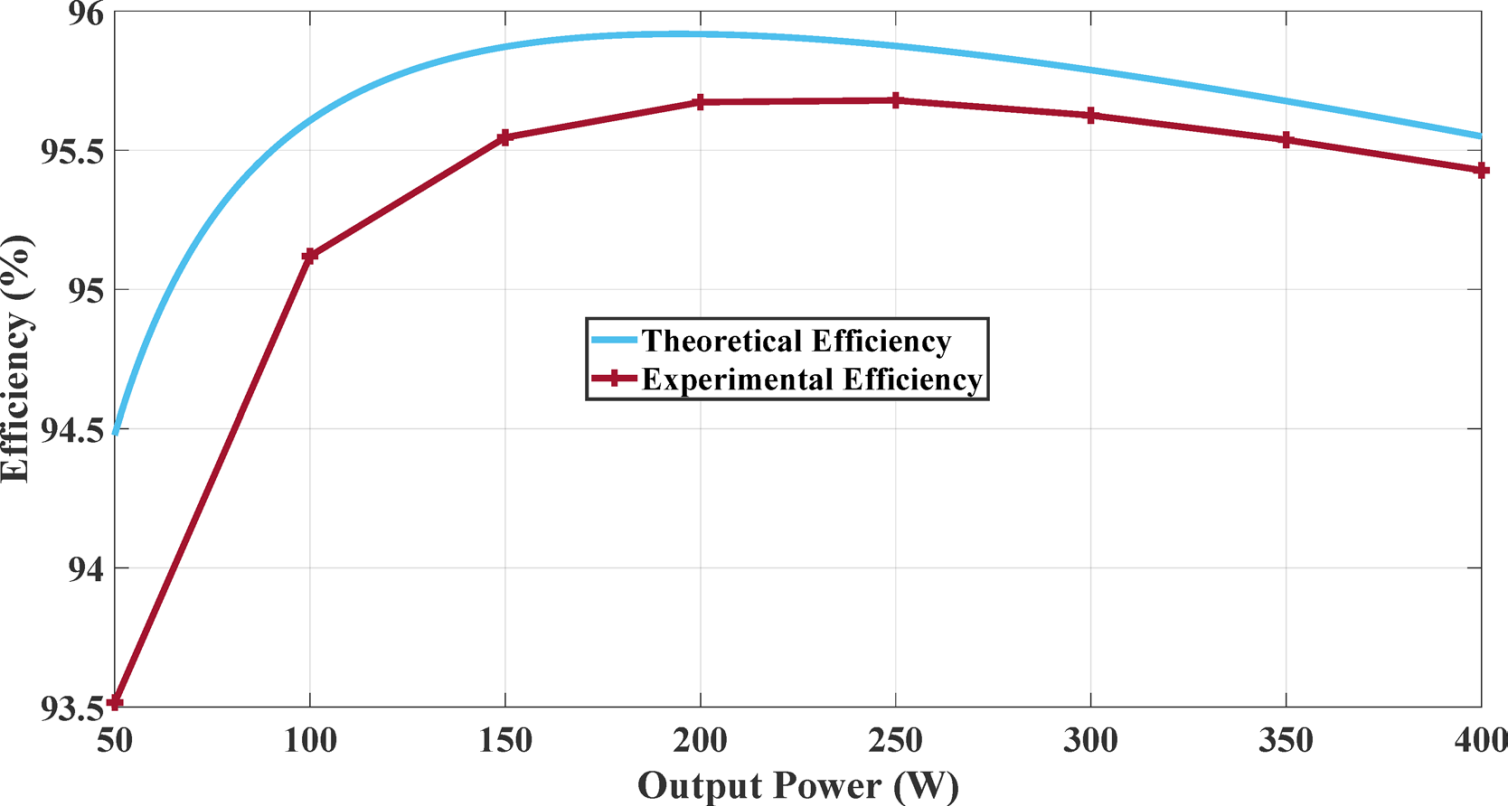
A detailed evaluation contrasting the predicted (theoretical) efficiency and the measured (experimental) efficiency of the proposed boost converter across different output power levels.

**Fig. 5:**
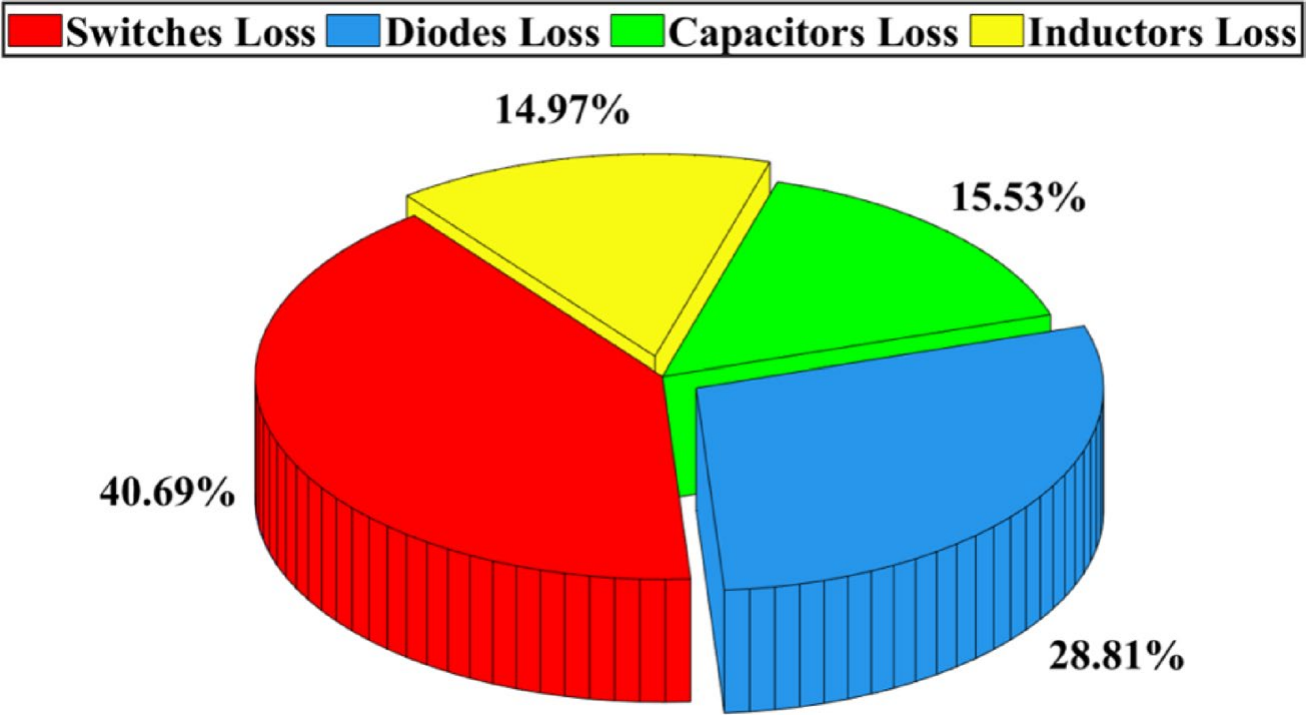
The proportional distribution of overall power losses attributed to the switches, diodes, capacitors, and inductors.

Figure 4 compares the analytically predicted efficiency of the proposed converter with experimentally measured efficiency as a function of output power. The theoretical efficiency curve is derived using the expressions presented in Eqs. (60)–(70). Furthermore, Fig. 5 provides a detailed distribution of power losses among the converter components, indicating that the majority of losses originate from the switching devices and diodes, while the contributions from the capacitors and inductors are comparatively small.

### Key parameter design guidance

#### Capacitors voltage ripple in boost mode

The capacitor values are determined by analyzing the average currents flowing through each capacitor across all switching states, while also considering the corresponding capacitor voltages, duty cycle, allowable voltage ripple (x_C_%), and the fixed switching frequency of 50 kHz. Using these design constraints, the minimum capacitance required for C_1_, C_2_, and C_O_ can be calculated as follows:

71 $${C_1} \geqslant \frac{{{I_O} \times (kn+1)}}{{{f_s} \times {V_{in}} \times {x_{C1}}\% }}$$

72 $${C_2} \geqslant \frac{{{I_O} \times (1 - D)}}{{{f_s} \times {V_{in}}(kn+1) \times {x_{C2}}\% }}$$

73 $${C_O} \geqslant \frac{{{I_O} \times D{{(1 - D)}^2}}}{{{f_s} \times (kn - D+2) \times {V_{in}} \times {x_{Co}}\% }}$$

#### Inductor design

Selecting appropriate values for the input inductor L and the magnetizing inductance L_m_ of the coupled inductor requires consideration of multiple key parameters. These include the average currents flowing through each inductor, the voltage levels applied during the various operating states, the duty cycle of the converter, the allowable current ripple limits (X_L_% for L and X_Lm_% for L_m_), and the switching frequency. Based on these design constraints, the minimum required inductance values for L and L_m_ can be expressed as follows:

74 $$L \geqslant \frac{{{V_{in}} \times D \times {{(1 - D)}^2}}}{{{f_s} \times {\mathrm{Io}}{\mkern 1mu} \left( {k{\mkern 1mu} n - D+2} \right) \times {x_L}\% }}$$

75 $${L_m} \geqslant \frac{{k \times {V_{in}} \times D}}{{{f_s} \times {\mathrm{Io}}{\mkern 1mu} \left( {n+1} \right) \times {x_{Lm}}\% }}$$

## Small-signal modeling

An appropriate pole-placement control strategy is introduced to ensure stable regulation of the output voltage. Designing an effective closed-loop control scheme necessitates the development of a small-signal model of the converter. Accordingly, the system equations corresponding to both switching subintervals are presented as follows:

76 $$\left[ {\begin{array}{*{20}{c}} {{{\dot {I}}_L}} \\ {{{\dot {I}}_{Lm}}} \\ {{{\dot {V}}_{C1}}} \\ {{{\dot {V}}_{C2}}} \\ {{{\dot {V}}_{Co}}} \end{array}} \right]=[{A_m}]\left[ {\begin{array}{*{20}{c}} {{I_L}} \\ {{I_{Lm}}} \\ {{V_{C1}}} \\ {{V_{C2}}} \\ {{V_{Co}}} \end{array}} \right]+[{B_m}]{V_{in}}$$

Where m = 1, 2.

By applying the averaging technique to the state-space equations and neglecting second-order perturbation terms, the small-signal representation of the proposed converter is obtained.

77 $$\left\{ \begin{gathered} \dot {\tilde {x}}=A\tilde {x}+B\tilde {u} \hfill \\ y=C\tilde {x}+D\tilde {u} \hfill \\ \end{gathered} \right.$$

The equations related to system states ($\tilde {x}$), control inputs ($\tilde {u}$), and the output (y) are presented as follows:

78 $${\tilde {x}^T}=\left[ {\begin{array}{*{20}{c}} {{{\tilde {i}}_L}}&{{{\tilde {i}}_{Lm}}}&{{{\tilde {v}}_{C1}}}&{{{\tilde {v}}_{C2}}}&{{{\tilde {v}}_{Co}}} \end{array}} \right]$$

79 $$\tilde {u}=\left[ {\tilde {d}} \right]$$

80 $${y^T}=\left[ {{V_{Co}}} \right]$$

Within the pole-placement control framework, the closed-loop poles can be assigned freely provided that the system is fully controllable in the state-space domain. Based on this requirement, the controllability matrix of the proposed converter is derived as follows:

81 $${\Phi _C}=\left[ {B \vdots AB \vdots {A^2}B \vdots \cdots \vdots {A^{n - 1}}B} \right]$$

A system is considered completely controllable when the rank of the controllability matrix equals five, matching the total number of state variables. To satisfy this condition, two additional integral state variables are introduced as follows:

82 $$\dot {q}(t)=r(t) - y(t)=r(t) - {\tilde {v}_{Co}}(t)$$

Following the inclusion of the integral state variables, the state-space and output equations are revised as follows:

83 $$\begin{gathered} \left[ {\begin{array}{*{20}{c}} {\dot {\tilde {x}}(t)} \\ \cdots \\ {\dot {q}(t)} \end{array}} \right]=\left[ {\begin{array}{*{20}{c}} A& \vdots &0 \\ \cdots & \vdots & \cdots \\ { - C}& \vdots &0 \end{array}} \right]\left[ {\begin{array}{*{20}{c}} {\tilde {x}(t)} \\ \cdots \\ {q(t)} \end{array}} \right]+\left[ {\begin{array}{*{20}{c}} B \\ \cdots \\ 0 \end{array}} \right]\tilde {u}(t)+\left[ {\begin{array}{*{20}{c}} 0 \\ \cdots \\ I \end{array}} \right]r(t) \hfill \\ y(t)=\left[ {\begin{array}{*{20}{c}} C& \vdots &0 \end{array}} \right]\left[ {\begin{array}{*{20}{c}} {\tilde {x}(t)} \\ \cdots \\ {q(t)} \end{array}} \right] \hfill \\ \end{gathered}$$

**Fig. 6:**
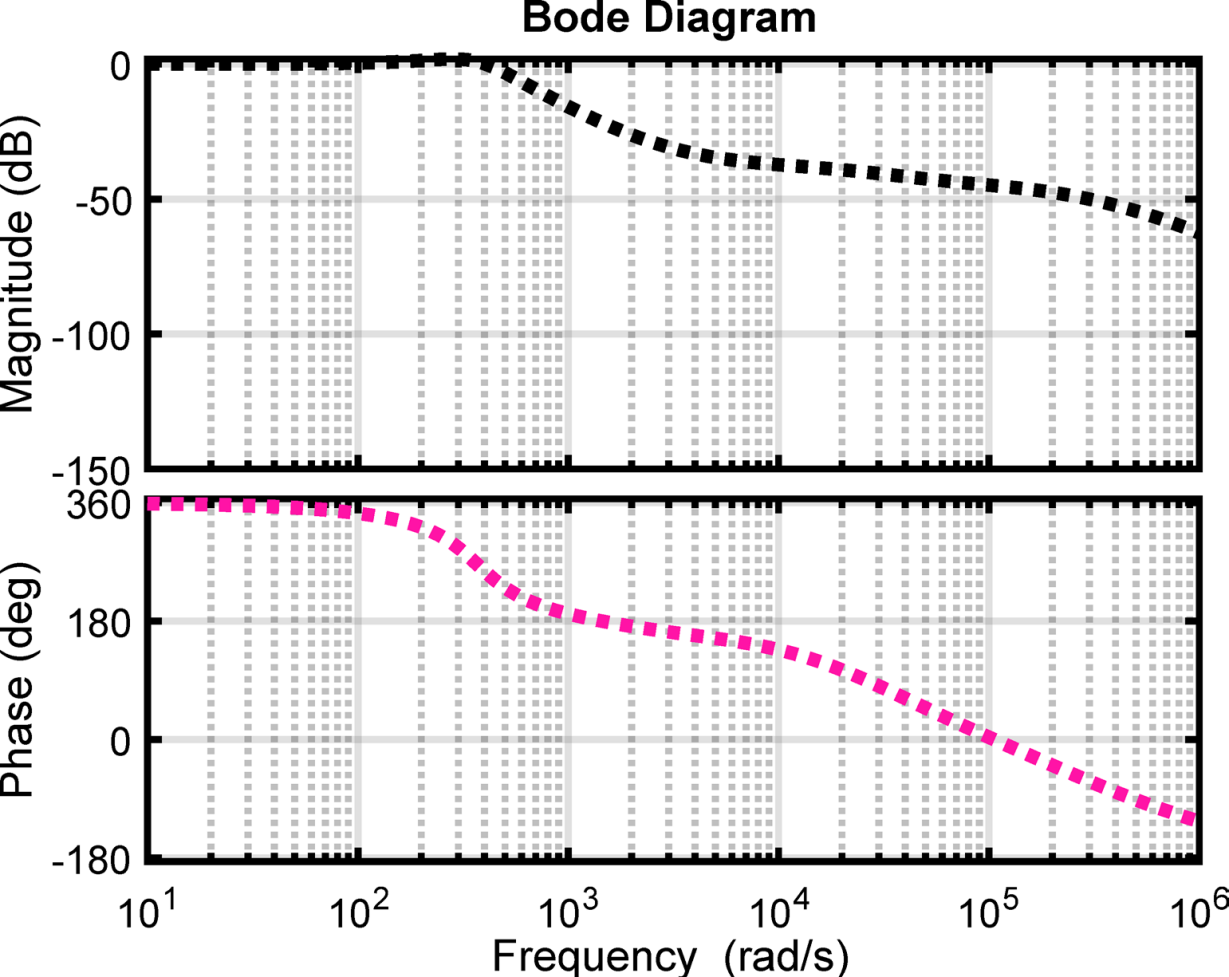
Bode plot of the transfer function associated with the output capacitor voltage.

**Fig. 7:**
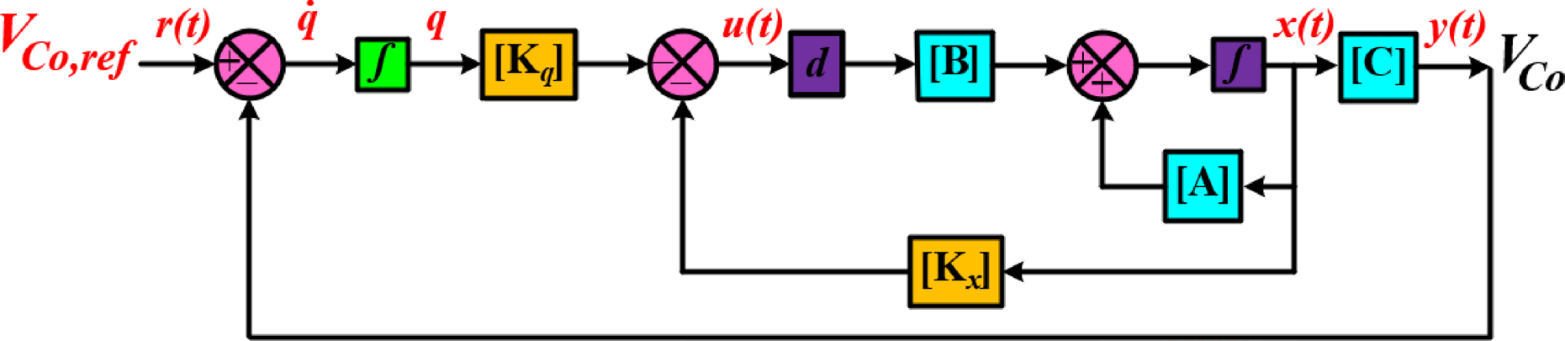
Block diagram of the pole-placement control method.

Provided that matrix is of full rank, the system described in (83) is regarded as completely controllable when the rank of matrix M equals n + m. To ensure adequate stability performance, the pole-placement controller is designed to achieve a gain margin (GM) of at least 10 and a phase margin (PM) within the range of 60° to 80°. A trial-and-error tuning procedure is employed to appropriately assign the closed-loop pole locations. Following this approach, the frequency response of the proposed control system is depicted in the Bode diagram shown in Fig. 6. The results indicate that the gain margin associated with the output capacitor voltage V_CO_ exceeds the required threshold, with GM (V_CO_) > 10. In addition, the corresponding phase margin of the closed-loop control loop regulating V_CO_ is calculated to be 74.51°, which lies well within the desired stability range. Moreover, Fig. 7 illustrates the block diagram of the implemented pole-placement control structure.

### Comparison study

A comprehensive comparative study was conducted to highlight the advantages of the proposed converter. In total, twelve recently published step-up topologies were examined, including four designs reported in 2025 and eight from 2024. Table 1 presents a detailed, criterion-by-criterion comparison between the proposed converter and these competing solutions, addressing key performance indicators such as voltage gain, maximum switch voltage stress, rated power, efficiency, total component count, and the availability of a common ground between input and output. The comparison is based on converters documented in references^11–22^.

**Fig. 8:**
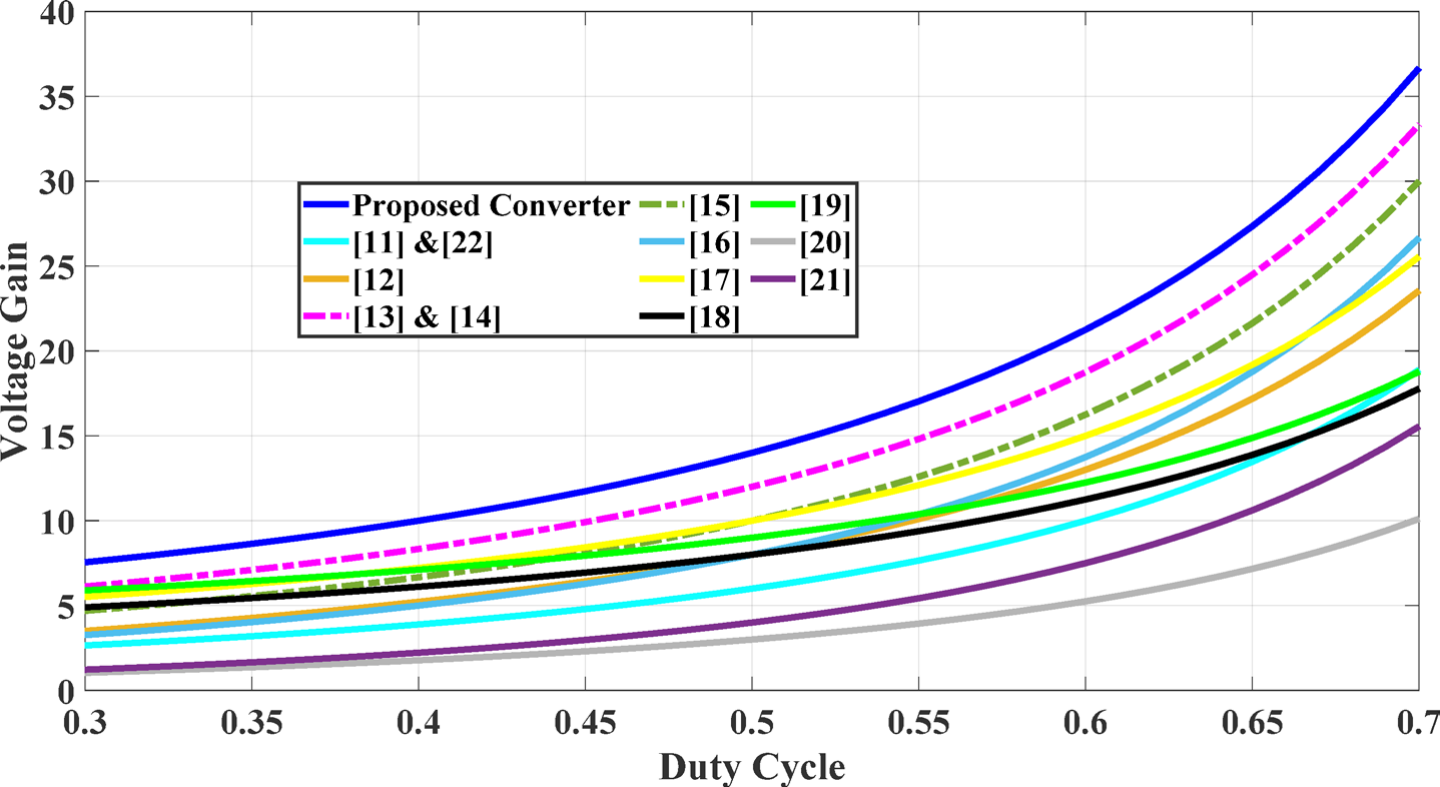
Variation of the output voltage gain with duty cycle for the compared high step-up converter topologies.

Given that the primary objective of this work is to achieve high step-up capability, voltage gain is treated as the dominant evaluation parameter. Using the numerical data summarized in Table 1; Fig. 8 illustrates the relationship between voltage gain and duty cycle for all investigated boost converters. The results clearly demonstrate that the proposed topology consistently attains a higher voltage gain than all other designs included in the comparison.

**Table 1:** Comparison between the propounded configuration and other circuits.

Ref/PY*	Voltage gain	Maximum voltage stress on Switches	Power (W) /Efficiency	No. of components*					Total component count /Common ground
				S	D	C	L	CI	
PC*	$\frac{{n - D+2}}{{{{(1 - D)}^2}}}$	$\frac{{{V_O}}}{{n - D+2}}$	400/95.55%	2	3	3	1	1	10/Yes
^11^ 2025	$\frac{{1+D}}{{{{(1 - D)}^2}}}$	${V_O}$	500/94.3%	2	3	3	2	0	10/No
^12^ 2025	$\frac{{(1+3D - 2{D^2})}}{{{{(1 - D)}^2}}}$	$\frac{{{V_O}}}{{1+3D - 2{D^2}}}$	100/96%	2	3	4	3	0	12/No
^13^ 2025	$\frac{{n+1}}{{{{(1 - D)}^2}}}$	$\frac{{{V_O}}}{{n+1}}$	360/95.2%	1	5	5	0	2	13/ Yes
^14^ 2025	$\frac{{n+1}}{{{{(1 - D)}^2}}}$	$\frac{{{V_O}}}{{n+1}}$	400/95%	1	4	4	2	1	12/ Yes
^15^ 2024	$\frac{{2+D}}{{{{(1 - D)}^2}}}$	$\frac{{{V_O}}}{{2+D}}$	50/87%	1	6	6	3	0	16/ Yes
^16^ 2024	$\frac{{2D+n - 1}}{{{{(1 - D)}^2}}}$	$\frac{{(1 - D){V_o}}}{{2D+n - 1}}$	210/93.4%	2	4	4	1	1	12/Yes
^17^ 2024	$\frac{{3 - D}}{{{{(1 - D)}^2}}}$	$\frac{{(1+2D - {D^2}){V_o}}}{{3 - D}}$	300/94.5%	1	6	4	3	0	14/Yes
^18^ 2024	$\frac{{3 - 2D}}{{{{(1 - D)}^2}}}$	$\frac{{(1 - 2D{{(1 - D)}^2}){V_o}}}{{3 - 2D}}$	300/93.6%	2	3	3	2	0	10/No
^20^ 2024	$\frac{{{{(2 - D)}^2}}}{{{{(1 - D)}^2}}}$	$\frac{{{V_o}}}{{{{(2 - D)}^2}}}$	400/93.3%	2	4	4	2	0	12/No
^20^ 2024	$\frac{{D(2 - D)}}{{{{(1 - D)}^2}}}$	$\frac{{{V_O}}}{{D(2 - D)}}$	72/95%	2	2	3	3	0	10/ Yes
^21^ 2024	$\frac{{2D}}{{{{(1 - D)}^2}}}$	$\frac{{(1+D){V_O}}}{{2D}}$	60/97%	2	3	4	3	0	12/ No
^22^ 2024	$\frac{{1+D}}{{{{(1 - D)}^2}}}$	$\frac{{{V_O}}}{{1+D}}$	100/94%	1	4	4	3	0	12/ Yes

*P.Y: Published year. *Number of Switches/Diodes/Capacitors/Inductors/Coupled Inductors.

**Fig. 9:**
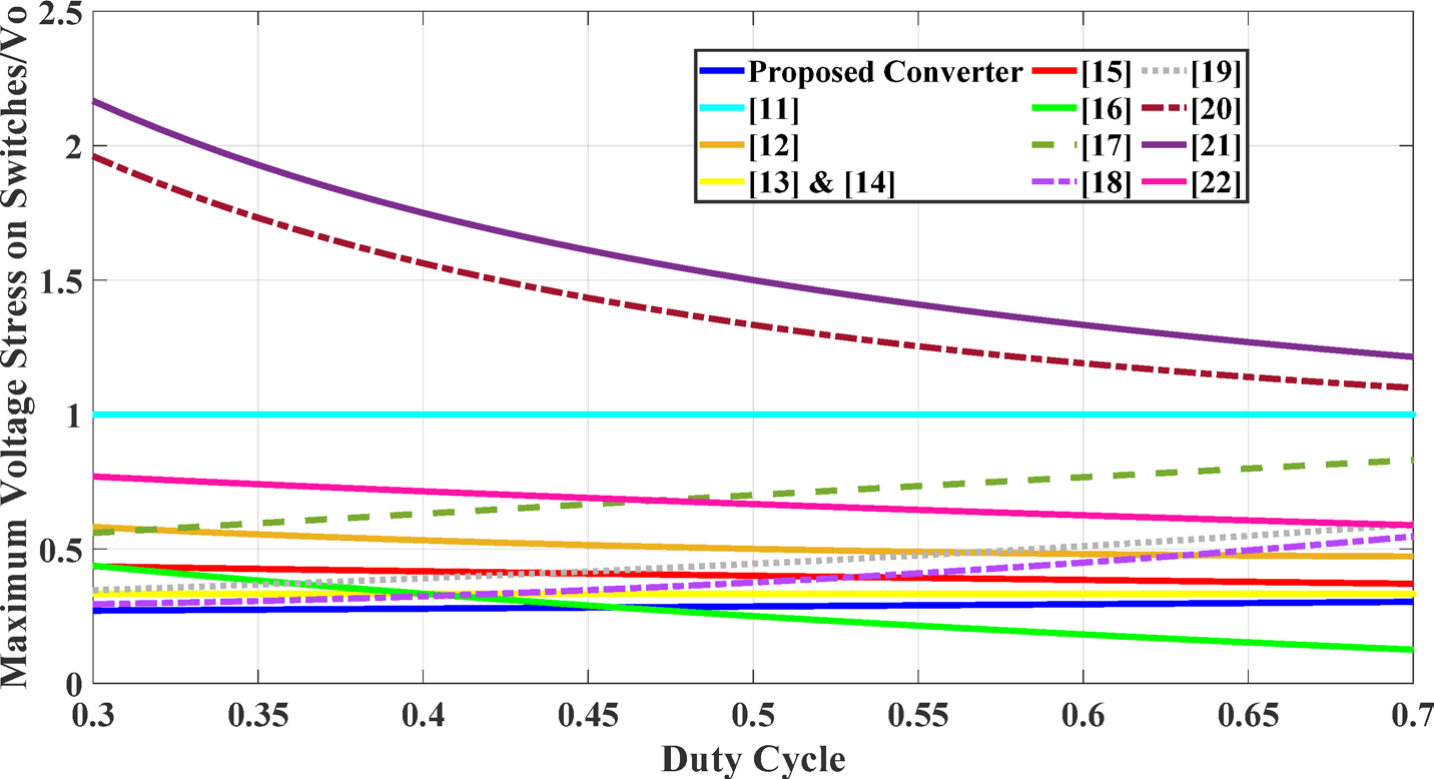
Comparison of maximum switch voltage stress as a function of duty cycle.

The second column of Table 1 lists the maximum voltage stress experienced by the switches, while Fig. 9 depicts how this stress varies with duty cycle. Over most of the operating range, the proposed converter maintains superior control over peak switch voltage stress when compared with alternative topologies. An exception is observed for the converter reported in^16^, where switch stress becomes lower than that of the proposed design when the duty cycle exceeds 50%. However, below this threshold, the proposed converter exhibits the lowest switch stress among all evaluated structures. Importantly, operation at duty cycles below 50% is generally preferred, as it improves converter performance and reduces losses due to the smaller duty ratio. Moreover, the relatively low voltage stress on the switches in the proposed topology allows for the use of smaller inductive components during the design process.

The third column of Table 1 focuses on rated power and efficiency. The proposed converter is designed to deliver 400 W and achieves an efficiency of 95.55%, as experimentally confirmed in Fig. 4. In contrast, most of the compared converters are rated for power levels below 400 W. Even at the full 400 W operating point, the proposed design maintains its high efficiency, surpassing that of the majority of competing topologies. Notably, several alternative designs operate at significantly lower power levels yet still exhibit inferior efficiency performance.

**Fig. 10:**
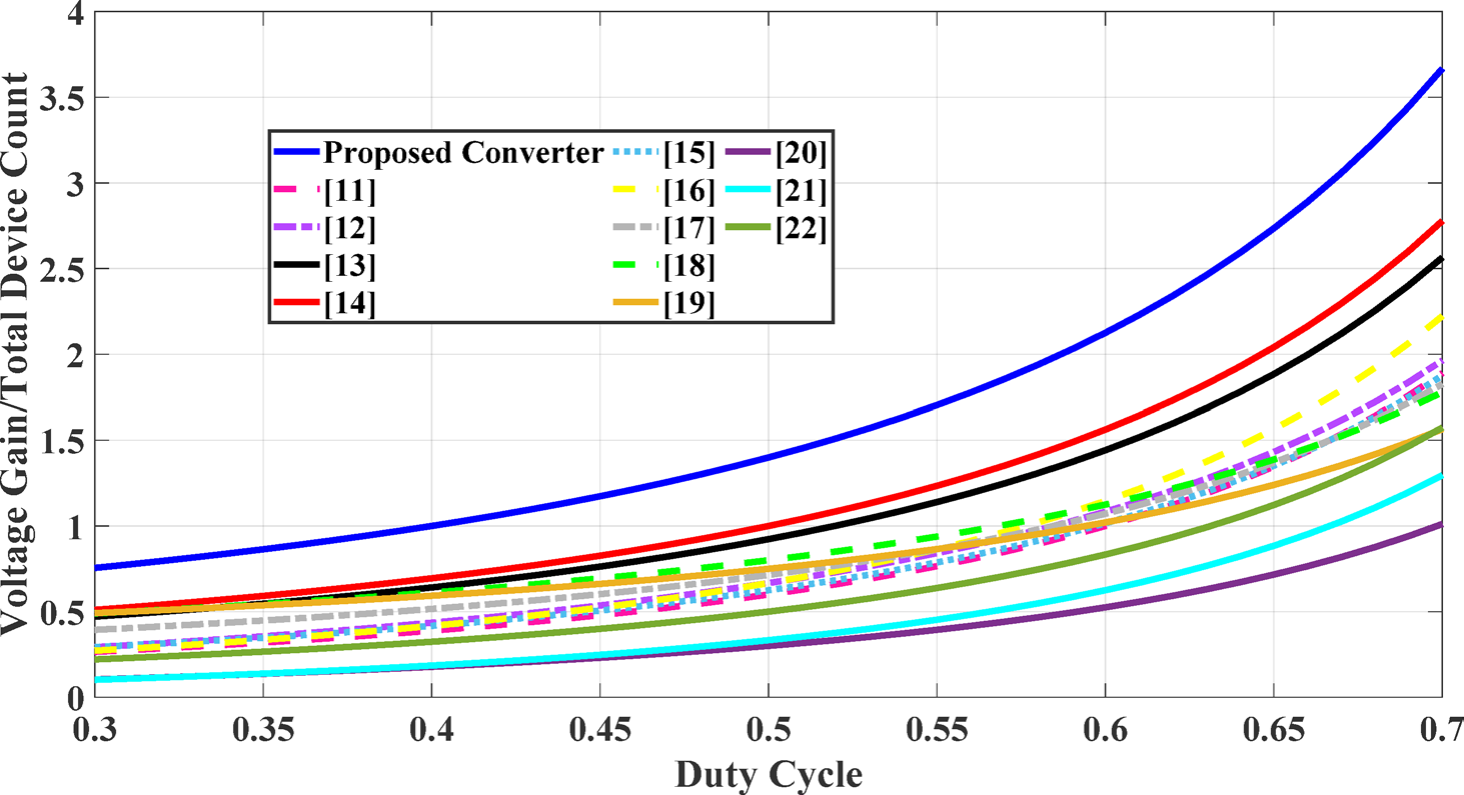
Ratio of voltage gain to total device count across different duty-cycle values.

Component utilization is summarized in the fourth column of Table 1, with the final column providing the total device count along with its breakdown. An effective high-gain converter should strike a balance between minimizing component count and maximizing voltage gain. To evaluate this trade-off, Fig. 10 plots voltage gain as a function of total device count for all considered configurations. The results indicate that the proposed topology achieves the highest voltage gain per total device count, suggesting a clear advantage in terms of cost-effectiveness for a given performance level.

Finally, the presence of a common ground between the input and output is examined, as this feature is critical in reducing electromagnetic interference and expanding applicability in practical systems. Topologies without a common ground are often more vulnerable to EMI-related issues, which can degrade performance and restrict their use. Unlike the converters reported in^11,12,18,19,21^, , the proposed design incorporates a shared ground reference. Overall, the comparative analysis confirms the strengths of the proposed converter, including superior voltage gain, reduced switch voltage stress, an outstanding gain-to-device-count ratio across duty cycles, and high efficiency at the 400 W power level.

## Experimental results

**Fig. 11:**
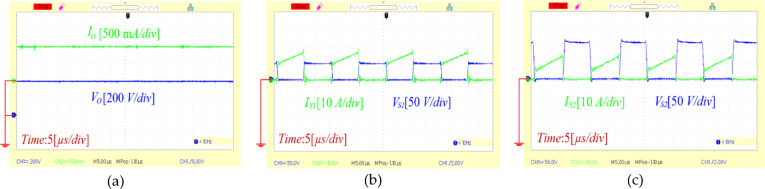
The experimental waveforms of output port and power switch, (**a**) voltage and current of the output port, (**b**) voltage and current of the power switch S_1_, (**c**) voltage and current of the power switch S_2_

To verify the accuracy of the analytical model and to confirm the practical feasibility of the proposed high–step-up converter, a 400 W experimental prototype was constructed in the laboratory. The principal design parameters of the prototype are listed in Table 2. Experimental testing demonstrates close agreement between measured results and theoretical analysis. As shown in Fig. 11a, the converter delivers an output voltage of 400 V with an output current of 1 A, which closely corresponds to the analytically predicted value of 406 V obtained from (26).

Figure 11b illustrates the voltage and current waveforms of switch S₁, revealing a peak voltage of 52 V and a current of 16 A values that are in strong agreement with theoretical expectations. Likewise, Fig. 11c shows that switch S₂ is subjected to a maximum voltage of 110 V and a current of 12 A, again matching the calculated results.

**Fig. 12:**
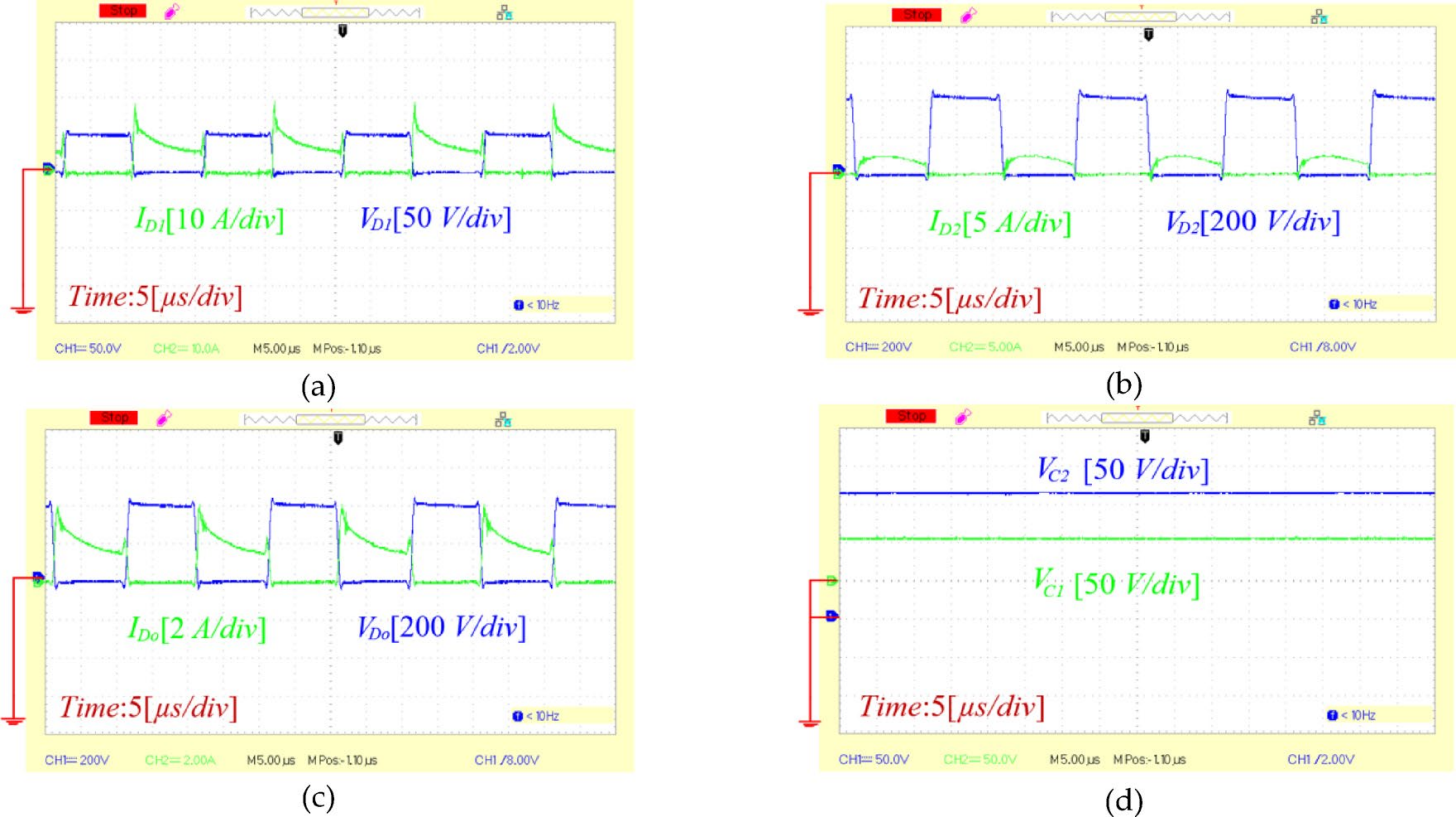
The experimental waveforms of diodes and capacitors, (**a**) voltage and current of the diode D_1_, (**b**) voltage and current of the diode D_2_, (**c**) voltage and current of the diode D_O_, (**d**) voltage across the capacitors C_1_ and C_2_

The experimentally measured voltage and current stresses across the diodes, along with the voltages across capacitors C₁ and C₂, are presented in Fig. 12. Specifically, Fig. 12a indicates that diode D₁ operates at approximately 51 V and 12 A, while Fig. 12b shows diode D₂ sustaining about 400 V and 3 A. As depicted in Fig. 12c, diode D_O_ experiences roughly 396 V and 3 A. The capacitor voltage measurements also show good consistency with theoretical predictions. Figure 12d reports voltages of 55 V across C₁ and 167 V across C₂, which are reasonably close to the analytically derived values of 58 V and 174 V obtained from (23) and (24).

Overall, the strong correspondence between the experimental waveforms shown in Figs. 11 and 12 and the analytical calculations confirms the correct operation of the proposed converter and substantiates its high-performance characteristics.

**Table 2:** Component characteristic of the implemented circuit.

Parameters	Values
Rated power (*P*_*o*_)	400 W
Input voltage	29 V
Output voltage	400 V
Switching Frequency (*f*_*s*_)	50 kHz
Turns Ratio *n* (*N*_*S*_*/N*_*P*_)	2
Input Inductor (*L*)	400 µH
Magnetizing Inductor (*L*_*m*_)	300 µH
Leakage Inductor (*L*_*k*_)	2 µH
Power Switches	IRFP260n
Diodes	Mur1560G
Cores type	ETD 49/25/16
Capacitor (*C*_1_)	68 µF/100V/ Electrolyte
Capacitor (*C*_2_)	68 µF/200V Electrolyte
Capacitor (*C*_***O***_)	100 µF/450V Electrolyte

## Conclusions

This work introduces an original non-isolated high step-up DC–DC converter that employs a coupled inductor and a reduced number of components to enhance both performance and efficiency. The proposed topology achieves a substantial increase in voltage gain by appropriately selecting the turns ratio of the coupled inductor, offering a simple yet powerful means of elevating the output voltage. One of the most important advantages of the design is its capability to keep the voltage stress on the power switches at relatively low levels significantly lower than those observed in many traditional high-gain converter topologies. As a result, low-voltage-rated switches with smaller on-state resistance can be utilized, which effectively reduces conduction losses and improves overall efficiency. The converter exhibits several key strengths, including a high voltage conversion ratio with limited power dissipation and flexible gain control achieved through both duty-cycle adjustment and the coupled-inductor turns ratio. In contrast to Z-source and quasi-Z-source converters, the proposed structure removes restrictions on the duty cycle, allowing operation across the entire range from 0 to 1. This capability enables the converter to reach very high voltage gains at relatively low duty cycles, thereby significantly decreasing conduction losses in the switching elements.

## Data Availability

All data generated or analyzed during this study are included in this published article.

## References

[1] Nadermohammadi, A. et al. Cost-effective quadratic ultra-high gain dc-dc converter with high power density for dc microgrid applications. *IEEE Open. J. Power Electron.*10.1109/OJPEL.2025.3614557

[2] Aghakhanlou, P. et al. New structure of step-up DC-DC converter based on three winding coupled inductor with high gain capability featuring integrated renewable energy applications. *Sci. Rep.* **14**, 31959 (2024). 39738419 10.1038/s41598-024-83502-4 PMC11686070

[3] Nadermohammadi, A. et al. Cost-effective soft-switching ultra-high step-up DC–DC converter with high power density for DC microgrid application. *Sci. Rep.* **14**, 20407. 10.1038/s41598-024-71436-w (2024). 39223195 10.1038/s41598-024-71436-w PMC11369153

[4] Hasanpour, S. A new trans-inverse coupled-inductor high step-up DC/DC converter with low voltage tensions. *IEEE Trans. Power Electron.*10.1109/TPEL.2025.3605789

[5] Rahimi, R., Habibi, S., Ferdowsi, M. & Shamsi, P. An interleaved quadratic high step-up DC-DC converter with coupled inductor. *IEEE Open. J. Power Electron.* **2**, 647–658 (2021).

[6] Gholizadeh, H. & Hasanpour, S. A new quadratic CUK-based step-up DC/DC converter without right hand plane zero. *Int. J. Ind. Electron. Control Optim.* **8** (1), 25–35. 10.22111/ieco.2024.48683.1565 (2025).

[7] Paşaoğlu, A. et al. A novel improved high-gain quadratic DC–DC converter based on two synchronous power switches and a two-winding coupled inductor. *Sci. Rep.*10.1038/s41598-026-50006-2 (2026). 42032100 10.1038/s41598-026-50006-2 PMC13275732

[8] Gholizadeh, H., Gorji, S. A. & Sera, D. A family of high-gain single-switch DC-DC converters for high-intensity discharge lamps. *IEEE Open. J. Power Electron.* **5**, 1534–1561 (2024).

[9] Sharma, P., Hasanpour, S. & Kumar, R. Ultra-high voltage gain achieved with quadratic DC/DC converter design. *Sci. Rep.* **14**, 23384. 10.1038/s41598-024-73984-7 (2024). 39379552 10.1038/s41598-024-73984-7 PMC11461893

[10] Nadermohammadi, A. et al. A non-isolated single-switch ultra-high step-up DC–DC converter with coupled inductor and low-voltage stress on switch. *IET Power Electron.* **17**, 251–265. 10.1049/pel2.12633 (2024).

[11] Naresh, S. V. K., Shareef, H., Kumar, B. & Peddapati, S. Family of capacitor–diode network extended high gain quadratic boost converters for microgrid applications. *IEEE Trans. Power Electron.* **40** (1), 1418–1430 (2025).

[12] Jayanthi, K. et al. Design and implementation of modified SEPIC DC–DC converter. *Electr. Eng.* **107**, 7873–7892. 10.1007/s00202-024-02934-3 (2025).

[13] Nadermohammadi, A., Seifi, A. & Hosseini, S. H. High-efficiency single-switch quadratic DC-DC converter for high-gain applications in integrated renewable energy systems. In *12th Iranian Conference on Renewable Energies and Distributed Generation (ICREDG), Qom, Islamic Republic of Iran, 2025* 1–6 (2025). 10.1109/ICREDG66184.2025.10966073

[14] Nadermohammadi, A. et al. Ultra-high step-up quadratic DC-DC converter with soft switching, high efficiency, and cost effectiveness for DC microgrid applications. In *16th Power Electronics, Drive Systems, and Technologies Conference (PEDSTC), Tabriz, Islamic Republic of Iran, 2025* 1–7 (2025). 10.1109/PEDSTC65486.2025.10911981

[15] Sumathy, P., Navamani, J. D. & Ali, M. Extendable high gain low current/high pulse modified quadratic–SEPIC converter for water treatment applications. *Sci. Rep.* **14**, 4899. 10.1038/s41598-024-55708-z (2024). 38418602 10.1038/s41598-024-55708-z PMC10901789

[16] Hasanpour, S. & Nouri, T. New coupled-inductor high-gain DC/DC converter with bipolar outputs. *IEEE Trans. Ind. Electron.* **71** (3), 2601–2613 (2024).

[17] Karthikkumar, S., Sheela, A., Talluri, M. T. & Krishna, B. Single switch hybrid network-based large step-up DC-DC converter for solar PV applications. *IEEE Trans. Circuits Syst. II Express Briefs*. **71** (7), 3573–3577 (2024).

[18] Lin, C. H., Khan, M. S., Ahmad, J., Liu, H. D. & Hsiao, T. C. Design and analysis of novel high-gain boost converter for renewable energy systems (RES). *IEEE Access.* **12**, 24262–24273 (2024).

[19] Samiullah, M., Hitmi, M. A. A., Iqbal, A. & Islam, S. Novel scalable topologies of high power density quadratic converters with low voltage stress on power diode. *IEEE Open. J. Ind. Electron. Soc.* **5**, 386–399 (2024).

[20] Okati, M., Eslami, M. & Khan, B. A novel semi-quadratic buck-boost structures with continuous input current for PV application. *Sci. Rep.* **14**, 14134. 10.1038/s41598-024-65012-5 (2024). 38898111 10.1038/s41598-024-65012-5 PMC11187186

[21] Hosseinpour, M., Heydarvand, M. & Azizkandi, M. E. A new positive output DC–DC buck–boost converter based on modified boost and ZETA converters. *Sci. Rep.* **14**, 20675. 10.1038/s41598-024-71612-y (2024). 39237808 10.1038/s41598-024-71612-y PMC11377812

[22] Diwakar Naik, M. & Vinatha, U. A novel single-switch high-gain DC–DC converter with active switched inductor. *IEEE Trans. Circuits Syst. II Express Briefs*. **71** (10), 4581–4585. (2024). 10.1109/TCSII.2024.3397010

[23] Nadermohammadi, A., Sorouri, H., Oshnoei, A., Hosseini, S. H. & Blaabjerg, F. Two-winding coupled-inductor-based DC–DC converter with two synchronous power switches and ultra-high voltage-gain capability. *Appl. Sci.* **16**, 1956. 10.3390/app16041956 (2026).

[24] Hasanpour, S. & Lee, S. S. A new quadratic DC/DC converter with ultrahigh voltage gain. *IEEE Trans. Power Electron.* **39**, 8800–8812. 10.1109/TPEL.2024.3385411 (2024).

[25] Nadermohammadi, A., Seifi, A. & Hosseini, S. H. Soft-switched high-gain Quasi-Z-source DC-DC converter with single magnetic core for dc microgrid applications. In *2025 16th Power Electronics, Drive Systems, and Technologies Conference (PEDSTC), Tabriz, Islamic Republic of Iran* 1–6 (2025).

[26] Abdi, M. et al. A high gain non-isolated DC-DC converter based on coupled-inductor with low voltage stress on switches for renewable energy applications. In *2024 9th International Conference on Technology and Energy Management (ICTEM), Behshar, Mazandaran, Islamic Republic of Iran* 1–6 (2024).

[27] Hajilou, M., Farzanehfard, H. & Vahedi, H. High step-up DC–DC converter with low switch voltage stress, continuous input current, and ZVS operation. *IEEE Open. J. Power Electron.* **6**, 277–285. 10.1109/OJPEL.2025.3532878 (2025).

[28] Aghakhanlou, P. et al. A single switch ultra-high step-up DC-DC converter based on a coupled inductor with two output ports for renewable energy applications. In *9th International Conference on Technology and Energy Management (ICTEM), Behshar, Mazandaran, Islamic Republic of Iran, 2024* 1–6 (2024). 10.1109/ICTEM60690.2024.10631942

[29] Hasanpour, S. & Nouri, T. A new quadratic step-up DC-DC converter with low voltage and current stresses. *Sci. Rep.* **15**, 38712. 10.1038/s41598-025-23433-w (2025). 41193631 10.1038/s41598-025-23433-w PMC12589525

[30] Ardakani, M. R. S., Hasanpour, S., Moazzami, M., Haghighatdar Fesharaki, F. & Hashemi, M. An improved cascaded boost topology according to the voltage lift technique with low voltage stress of components. *IEEE Access.* **13**, 188892–188906. (2025). 10.1109/ACCESS.2025.3626127

[31] Ndermohammadi, A. et al. A three-winding coupled inductor-based three-port ultra-high step-up DC-DC converter for renewable energy applications. In *9th International Conference on Technology and Energy Management (ICTEM), Behshar, Mazandaran, Islamic Republic of Iran, 2024* 1–6 (2024). 10.1109/ICTEM60690.2024.10631903

[32] Nadermohammadi, A., Hosseini, S. H. & Sabahi, M. A quadratic high-gain DC–DC converter integrating a two-winding coupled inductor with low voltage stress on both switches for renewable energy applications. *Sci. Rep.*10.1038/s41598-026-49207-6 (2026). 42049864 10.1038/s41598-026-49207-6 PMC13314953

[33] Hasanpour, S., Siwakoti, Y. & Blaabjerg, F. New single-switch quadratic boost DC/DC converter with low voltage stress for renewable energy applications. *IET Power Electron.* **13** (19), 4592–4600 (2020).

[34] Hosseini, S. J. A. et al. A new ultra-high voltage gain DC/DC converter based on coupled-inductor. *Sci. Rep.* **15**, 5330 (2025). 39948267 10.1038/s41598-025-90093-1 PMC11825954

[35] Abdi, H. et al. High-stability and cost-effective ultra high step-up DC-DC converter for solar-powered DC microgrids. *TABRIZ J. Electr. Eng.*10.22034/tjee.2025.66169.4979 (2025).

[36] Ndermohammadi, A. et al. A high-gain common-ground single-switch DCDC converter with low voltage stress on the power switch and diodes. In *2024 9th International Conference on Technology and Energy Management (ICTEM), Behshar, Mazandaran, Islamic Republic of Iran* 1–6 (2024).

[37] Aghakhanlou, P. et al. A high voltage gain quadratic boost DC-DC cConverter utilizing coupled-inductor with low voltage stress. In *16th Power Electronics, Drive Systems, and Technologies Conference (PEDSTC), Tabriz, Islamic Republic of Iran, 2025* 1–6 (2025). 10.1109/PEDSTC65486.2025.10911966

[38] Hasanpour, S. & Lee, S. S. A new coupled-inductor-based quadratic DC/DC converter with low current stress. *Energy Sci. Eng.* **13**, 1935–1947. 10.1002/ese3.70017 (2025).

